# Exploring the role of CBT in the self-management of type 2 diabetes: A rapid review

**DOI:** 10.4102/hsag.v28i0.2254

**Published:** 2023-05-29

**Authors:** Elne Visagie, Elmari Deacon, Rümando Kok

**Affiliations:** 1 Compress Research Focus Area, Faculty of Health Sciences, North-West University, Potchefstroom, South Africa; 2 Optentia Research Unit, Faculty of Health Sciences, North-West University, Potchefstroom, South Africa; 3 Centre for Health and Human Performance (CHHP), Faculty of Health Sciences, North-West University, Potchefstroom, South Africa

**Keywords:** type 2 diabetes, T2DM, cognitive behaviour therapy, self-management, adults

## Abstract

**Background:**

Type 2 diabetes has been recognised as a global health concern: one that requires intervention to lessen the incumbrance caused by the chronic illness. This rapid review was conducted to determine the scientific evidence available on how Cognitive Behaviour Therapy (CBT) interventions improved the self-management of individuals with type 2 diabetes.

**Aim:**

The aim of the review was to synthesise current scientific evidence regarding CBT-based interventions and self-management practices.

**Method:**

The rapid review served as a framework to appraise current national and international literature. The researchers used Google Scholar, Journal Storage (JSTOR), PsycINFO, APA PsycArticles, SAGE journals and EBSCO Discovery Services to search for relevant studies. This was performed by employing keywords. Nine relevant studies were identified. The studies were heterogenous in methodology. Seven of the nine studies were conducted in developing countries.

**Results:**

The study found that the context of developmental countries plays a significant role in the development of type 2 diabetes and requires tailored intervention because of socio-economic variabilities. The main themes identified in relation to improving self-management included: the characteristics of the CBT-based interventions, namely the format, duration, and outcomes, and identifying the techniques and components used in the CBT-based interventions.

**Conclusion:**

The review emphasised the need to further investigate the role of CBT in improving self-management of type 2 diabetes, especially in a South African context.

**Contribution:**

The review summarised the techniques that have proven to be effective for the self-management of type 2 diabetes.

## Introduction

Diabetes mellitus has become a global crisis, with approximately 537 million individuals having been diagnosed with diabetes. Over 90% of these cases are represented by type 2 diabetes mellitus (T2DM) (Asiimwe, Mauti & Kiconco 2020). The incidence of T2DM has also been increasing rapidly in developing countries, with approximately 80% of adults with diabetes living in developing countries (Lin et al. 2020). It is a diagnosis that carries significant implications regarding the health, finances, social relatedness, and personal well-being of individuals (Unnikrishnan et al. 2017). The average age of diagnosis of 45 years necessitates numerous lifestyle changes during a time when individuals are settled in their routines (Zoungas et al. 2014). In developing countries, this adjustment is further complicated by a change in food consumption patterns, reduced physical activity, a lack of knowledge regarding diabetes, poor access to health systems, migration, urbanisation, and under-resourced healthcare systems (Misra et al. 2019). Within these settings, the self-management of T2DM is suboptimal because of the inaccessibility of medication, knowledge, and interventions (Misra et al. 2019). This highlights the need for time-efficient and cost-effective interventions. This need is caused by the financial burden of managing T2DM, in addition to under-resourced settings (Lin et al. 2020). As self-management is the cornerstone of diabetes management, it is imperative to explore and develop more cost-effective strategies for individuals, specifically in developing countries (Van Smoorenburg et al. 2019).

Self-management is the foundation of diabetes management because individuals actively participate in their care plans and treatment (Voeltz et al. 2022). Self-management includes three distinctive tasks: medical management (medication adherence), behaviour management (changing behaviours and adopting new management behaviours), and emotional management (managing diabetes distress) (Van Smoorenburg et al. 2019). Strategies and interventions focusing on improving T2DM self-management have been linked to improved health outcomes and quality of life (Uchendu & Blake 2017). However, self-management practices remain challenging in most countries, as approximately 60% of individuals struggle with self-management and glycaemic control (Yang, Li & Sun 2020).

How an individual with T2DM copes with the thoughts, emotions, and behaviours relating to self-management can intensify or alleviate the burden of managing T2DM (Van Smoorenburg et al. 2019). Identifying and modifying beliefs and behaviours that hinder successful self-management can facilitate a change in thought patterns and improve self-management practices (Carpenter, DiChiacchio & Barker 2019). It is proposed that cognitive behaviour therapy (CBT) can be an effective intervention for improving self-management in individuals with T2DM. Cognitive behaviour therapy postulates an interaction between our thoughts, emotions, and behaviour (Beck 1976). It enables individuals to identify automatic negative thoughts, reorganise and reframe these thought patterns, manage emotions, and change unhealthy behaviours (Yang et al. 2020). Cognitive behaviour therapy interventions regarding T2DM self-management aim to make individuals aware of how automatic negative thoughts affect their self-management practices and how to modify thought and behaviour patterns to improve self-efficacy (Kanapathy & Bogle 2019). Being aware of this relationship and developing the skills to adapt cognitive, emotional, and behavioural responses can assist individuals in attaining a satisfactory quality of life (Barlow et al. 2002).

Research predominately focuses on how CBT improves mood disorders in individuals with type 2 diabetes (Clarke et al. 2019; Cummings et al. 2019). Few studies focus on how CBT can be used to improve self-management practices directly, regardless of mood disorders being present. Exploring the thoughts, emotions and behaviours relating to self-management practices can enable individuals to identify where they need to adapt the thoughts and behaviours that act as barriers to the effective self-management of their T2DM (Martz 2017).

This study aimed to fill this research gap by performing a rapid review to synthesise current scientific evidence regarding CBT-based interventions and self-management practices. Therefore, the current rapid review intended to examine peer-reviewed studies using CBT, group CBT or combined CBT that were administered face-to-face or via an online platform to increase the understanding of the efficacy of CBT in individuals with T2DM.

## Methodology

A rapid review of current international and national literature on T2DM self-management and CBT interventions was conducted. A rapid review is rigorous and adheres to the core principles of a traditional systematic review to avoid prejudice during the research process (Schünemann & Moja 2015). Rapid reviews aim to promptly synthesise knowledge from various sources (Tricco et al. 2015). A rapid review follows the key principles of a systematic review, but rapid reviews differ in that the process is accelerated to produce information promptly (Shigekawa et al. 2018). It assesses what is already known regarding practice and policy matters and balances an expedited process with the necessary methodological rigour to deliver transparent and formal quality assessments (Moons, Goossens & Thompson 2021). This study followed the framework by Dobbins (2017), which set out five steps to conduct a rapid review and extract data from the nine studies. The five steps included: (1) defining the practice question, (2) searching for research evidence, (3) critically appraising the information, (4) synthesising the evidence, and (5) identifying applicability and transferability issues for further consideration during the decision-making process (Dobbins 2017).

During step one, the practice question was identified. This rapid review aimed to answer the following research questions:

- What CBT interventions address self-management practices for individuals with T2DM?
- What techniques and components are used in the CBT intervention to address self-management practices?

### Search strategy

Step 2 involved searching for research evidence. Searching for research evidence included the search for peer-reviewed journals by using Google Scholar, Journal Storage (JSTOR), PsycINFO, APA PsycArticles, SAGE journals and EBSCO Discovery Services, which were accessible from the North-West University library portal. Specific keyword formulations were used to search the titles, abstracts, and texts of the journal articles. Boolean operators (such as AND, NOT, OR) were used in conjunction with the following keywords to search:

In the abstract and title: ‘type 2 diabetes’ OR ‘T2DM’ AND ‘cognitive behavioural therapy’ OR ‘cognitive behavioural therapy’ OR ‘CBT’ AND ‘Adult’ OR ‘patient’ AND ‘self-management’ OR ‘self-care’ OR ‘self-regulation’.

Peer-reviewed studies, including quantitative, qualitative, mixed-method or multimethod studies published between 2012 and 2022 were included in the review. Studies had to focus on how CBT interventions aided self-management practices in adults with T2DM. Studies that focused on type 1 diabetes or solely on the effect of CBT on depression in adults with T2DM were excluded. Studies that were not published in English were also excluded.

**FIGURE 1:**
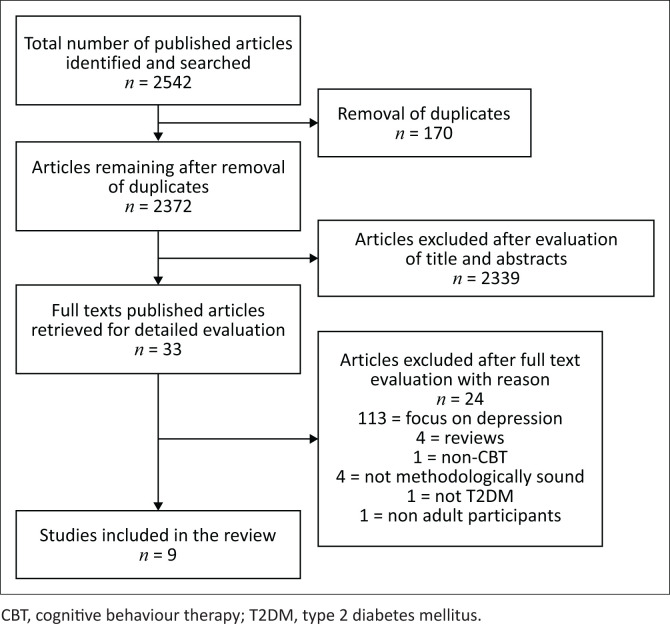
Search flow chart: Realisation of the search strategy.

Step three commenced and involved critically appraising the information. The three reviewers independently screened the studies based on the above-mentioned inclusion and exclusion criteria (*n* = 2542). After the duplicates had been removed (*n* = 170), the researchers screened the titles of the studies, and 2339 articles were removed. The abstracts of the remaining 33 articles were screened and all were deemed relevant. The full text of the remaining studies (*n* = 33) was screened. After screening the complete text, 18 studies were removed because they did not meet the inclusion and exclusion criteria. Of the 18 studies excluded, 13 were excluded because of focusing specifically on depression, four were excluded as a result of being systematic reviews, and one was excluded because of using a combination of CBT and a different therapeutic approach. Fifteen studies were reviewed according to the JBI Critical Appraisal Checklist for quantitative and qualitative studies to determine scientific quality. Upon appraising the text, one study was found to focus on type 1 and type 2 diabetes, one study had non-adult participants, and four studies were found to be not methodologically sound. This referred to studies that did not fulfil the JBI Critical Appraisal Checklist and demonstrated incongruence between the research methodology and the methods used to collect data. Therefore, these studies were excluded from the review. Nine studies were identified to be included in the rapid review. Figure 1 represents a schematic overview of the screening process which formed part of the critical appraisal phase.

### Data analysis

Step 4 and 5, data extraction and synthesis, took place after the critical appraisal of the data. The data were analysed by means of thematic synthesis. Thematic synthesis involves systematic coding, generating descriptive themes and developing analytical themes (Nicholson et al. 2016). Firstly, the text was coded according to a number system. The content was coded according to its meaning and subject matter (Nicholson et al. 2016). Secondly, grouping codes were used to develop descriptive themes according to similarities (Nicholson et al. 2016). Thirdly, analytical themes were derived from the descriptive themes to identify new explanations that could answer the review questions. Table 1 summarises the data extracted from the nine articles.

**TABLE 1:** Data extraction of nine eligible studies.

Number	Author(s) and title	Research design and aim	Sample characteristic	Measurements	Intervention	Findings
1	Ardeshirpey et al. (2021) Effect of MBCT on cognitive emotion regulation, perseverative thinking, and glycaemic index in patients with type 2 diabetes A trial study *Iranian Journal of Diabetes and Obesity*	Quasi-experimental The study aimed to determine how participation in an MBCT programme affected emotional and cognitive regulation, glycaemic control, and preservative thinking.	Iran Males and females 30 to 60 years Experimental group: *n* = 15 Control group: *n* = 15	Emotion Regulation Questionnaire, Perseverative Thinking Questionnaire, and an FBS test	Nature of the intervention: Mindfulness-based cognitive therapy Group-based Eight 90-min group sessions The sessions were held over 2 months The sessions were based on two books, namely, *The mindful way through depression* Williams et al. 2007 and *Mindfulness-based cognitive therapy* (Barnhofer et al. 2009) No information on who led the groups Participants were pre-tested before the start of the research Post-tests were performed upon completion of the eight sessions Components of the intervention: Establishing rapport and providing psychoeducation on the relationship between thoughts, emotions, and behaviour Emotional regulation (monitor, evaluate and modify beliefs) Cognitive restructuring (re-assess current cognitive assessment of their lives and situation Identifying and modifying cognitive distortions Disengaging from thoughts and emotions that are interpreted as negative Managing and disengaging from intrusive thoughts Enhancing the ability to endure uncomfortable emotional states Enhancing an individual’s ability to receive unpleasant thoughts and emotions without severe emotional reactions The aim is to change the relationship the individual has with uncomfortable emotions and thoughts Reduce the avoidance of unpleasant emotions by practising mindfulness and acceptance Homework assignments Creating an action plan for future difficulties, evaluation of the programme by the members The control group: Received no intervention	Improved cognitive and emotional regulation strategies Improved glycaemic control Improved perseverative thinking skills
2	Empraninta, Purba and Asrizal (2021) The effect of cognitive behavioural therapy on the self-management and self-care behaviour of T2DM patients *Jurnal Keperawatan Soedirman*	Quasi-experimental The research aimed to determine how CBT affected self-management behaviours in individuals with T2DM.	Indonesia Males and females Aged between 46 and 65 years Intervention group: *n* = 35 Control group: *n* = 35	The DSMQ, SDSCA	Nature of the intervention: Cognitive behaviour therapy Five sessions were conducted over the course of 4 weeks Each session was 30 min to an hour in duration Researchers performed the intervention Components of the intervention: Psychoeducation regarding the cognitive-behaviour model Identifying and changing maladaptive thoughts Role-play Homework The control group: Received no psychological intervention	The study found that CBT had a positive effect on self-management and self-care behaviours Improved behaviours and attitudes towards T2DM
3	Inouye et al. (2015) Psychosocial and clinical outcomes of a cognitive behavioural therapy for Asians and Pacific Islanders with type 2 diabetes: A randomized clinical trial *Hawai’i Journal of Medicine & Public Health*	Randomised controlled clinical trial The study aimed to determine how participation in CBT affected glycaemic control, quality of life, health perceptions and mood-related symptoms.	Hawaii Males and females 18- to 76-year-olds *n* = 207	Diabetes Quality of Life Survey, Medical Outcome Study SF-36 Survey, CES-D scale, SDSCA Questionnaire, MDQ, and the Health Belief Scale	Nature of the intervention: Cognitive behaviour therapy and diabetes education (in a group setting) Six sessions over 6 weeks (one per week) Each session averaged 1 to 2 h Research assistants led the sessions The research assistants received training in CBT from the investigator Fidelity checks were performed at several intervals (with the participants’ consent) During the participation of the sessions, friends and family members were encouraged to accompany participants and participate Pre-tests of psychosocial and clinical outcomes were completed before the sessions commenced Post-tests were conducted upon completion of the sessions and again after 12 months post-sessions. The researchers recommended booster sessions for improved results and to assist and facilitate individuals with better long-term outcomes and benefits Components of the intervention: Monitoring diabetes management and setting goals Modifying behaviours and habits/behavioural rehearsal Introducing and implementing cognitive restructuring techniques to modify maladaptive thoughts Managing stress Social support Relaxation techniques Mood management Self-efficacy and empowerment Improving problem-solving and decision-making skills Reviewing skills and practices at the start of each session Giving homework assignments The control group: Received DES Received 6 weekly sessions that averaged 1 to 2 h Each group had two to six participants The goal was for participants to share personal experiences and review diabetes education Different research assistants led the sessions. The research assistants received training from the investigator Themes covered in the discussions included: insurance coverage, dental care, foot care, managing sick days, and taking your diabetes on holiday	Short-term improvements that were noticed included: Improved glycaemic control Improved quality of life Improved perceptions of health Reduced symptoms of low mood Improved adherence to self-management According to the research, these changes were short-lived and were not present after a 12-month follow-up.
4	Mousavian, Mujembari and Aghayousefi (2018) The effectiveness of cognitive therapy on quality of life in patients with type 2 diabetes *International Archives of Health Sciences*	Semi‑experimental with pre-test/post-test/follow‑up and control group The study aimed to determine the effect of cognitive therapy on the quality of life.	Iran Adults Males and females 25 to 45 years old Experimental group: *n* = 20 Control group: *n* = 20	The DQOL Survey (Measurement D-39)	Nature of the intervention: Cognitive therapy intervention Ten sessions over 10 weeks (one session per week) Each session was approximately 2 h No information on who led the groups Pre-tests were conducted before the therapeutic intervention Post-tests were conducted after the therapeutic intervention, and there was a follow-up after 3 months Components of the intervention: Establishing rapport Psychoeducation about cognitive therapy Identifying and understanding automatic thoughts and identifying common themes in automatic thoughts Identifying and modifying unhelpful cognitive processes (cognitive restructuring) by identifying cognitive distortions and using Socratic questioning, a thought record and the evidence technique Identifying and implementing alternative thoughts and behaviour Relaxation strategies Emotional regulation Giving homework assignments Devising an action plan for continuing the learned techniques The control group: Did not receive any therapeutic intervention	Participation in the intervention led to a perceived improvement in quality of life A change of attitude towards the self-management of type 2 diabetes In addition, improved quality of life was due to a more flexible approach to self-management and lifestyle Follow-up at 3 months reported continuous improvement in quality of life
5	Pan et al. (2020) A group-based community reinforcement approach of cognitive behavioural therapy programme to improve self-care behaviour of patients with type 2 diabetes *Frontiers in Psychiatry*	Prospective interventiontrial The research objective was to evaluate the effect of a cognitive behavioural-based self-care intervention on type 2 diabetes self-management.	China Adults Males and females 18 to 70 years old Intervention group: *n* = 296 Control group: *n* = 110	Body weight and blood pressure were checked at baseline, after 12 weeks, and after 24 weeks. A biomedical assessment that assessed HbA1C levels. Diabetes knowledge scale. A questionnaire was developed for the study that collected information relating to the participants’ demographics, food consumption, physical activity (body weight, blood pressure, HbA1C), medical history, knowledge of self-care behaviours, and health knowledge. The SF-12 Quality of Life questionnaire, Self-efficacy scale, self-reported questionnaire An assessment of the participants’ satisfaction with the programme was administered (after the intervention)	Nature of the intervention: Intensive cognitive behaviour-based self-care intervention (group-based) Twelve sessions; one session per week Each session was 60 to 90 min long and consisted of 10 to 15 participants. Experienced, community-based physicians led the sessions An experienced psychologist trained the physicians The participants received a list of low glycaemic foods and healthy eating instructions (based on the Chinese health diet plan) During the intervention, participants consulted with a community physician regarding their diet and for medical evaluation. The physician reviewed the participants’ medication and adherence to healthy practices and measured and assessed their blood glucose. During the intervention period, the participants monitored their HbA1c, started an exercise programme, managed their foot care, and consumed healthier foods. Participants recorded their daily self-care behaviours, the problems they encountered, and the symptoms they experienced in a diary During the sessions, participation was recorded by the community physicians Components of the intervention: Building rapport Psychoeducation regarding the relationship between thoughts, emotions and behaviours about type 2 diabetes Cognitive restructuring Socratic questioning to identify maladaptive thoughts Developing and implementing alternative thoughts Identify and modify beliefs about efficacy regarding glycaemic control Identify and modify behaviours to improve self-management Practising acceptance of self-management Identifying new coping skills Self-regulation skills Self-reflection skills Lifestyle modification (keeping a journal of challenging thoughts, behaviours, and situations and writing down alternative reactions) Implementing self-care practices Social support Homework assignments The control group: The control group received diabetes education that included education on diet and physical activity	The control group who received the intervention experienced the following outcomes with regard to self-management: Reduced blood pressure Reduced HbA1c Improved knowledge regarding diabetes self-care Modified behaviour with regard to T2DM Improved general health Follow-up at 6 months indicated improved glycaemic control The study concluded that a multi-dimensional-based cognitive behavioural intervention focuses on psychoeducation, self-monitoring and exercise. Self-care and behavioural skills can improve diabetes self-management.
6	Seyed-Reza, Norzarina and Kimura (2015a) Effect of group CBT on psychological well-being and glycaemic control in adults with type 2 diabetes *International Journal of Diabetes in Developing Countries*	Experimental The research aimed to determine whether participants’ glycaemic control, self-management and psychological well-being would improve if they participated in group CBT.	Malaysia Males and females 20 to 65 years Individuals of various ethnic backgrounds Experimental group: *n* = 30 Control group: *n* = 30	HbA1C and the W-BQ 22	Nature of the intervention: Group CBT Eight sessions over 12 weeks The first four sessions were held weekly and the second four sessions were held every second week A psychologist led the sessions Participants were encouraged to work through the daily challenges they experienced The participants had to record the challenges that they encountered Before the sessions took place, the participants were given a detailed description of group CBT Participants were pre-tested before the sessions began Post-tests were conducted after the sessions concluded (at the end of the 3 months) Components of the intervention: Monitoring of diabetes-related difficulties and feedback Psychoeducation Identifying sources of stress and triggers Developing an awareness of participants’ thoughts, emotions and actions Identifying irrational thoughts and restructuring thought processes Adapting behaviours Exploring alternative coping strategies Identifying the emotions Improved decision-making Social support in the group setting The control group: Did not receive any psychological interventions.	Improved glycaemic control after the intervention Improved self-management because of improved self-efficacy and problem-solving skills Increased psychological well-being The research highlighted the need to explore and integrate cognitive models into diabetes care plans
7	Seyed-Reza, Norzarina, and Kimura (2015b) The benefits of CBT on diabetes distress and glycaemic control in type 2 diabetes *Malaysian Journal of Psychiatry*	Quasi-experimental The research objective was to determine how effective group CBT was in improving glycaemic control and diabetes distress.	Malaysia Males and females 30- to 65-year-olds Experimental group: *n* = 30 Control group: *n* = 30	HbA1c and the DDS-17	Nature of the intervention: Group CBT Eight sessions over three months (weekly for the first month and after that every second week) The sessions were led by a psychologist (there were three experimental groups, each consisting of 10 participants) The pre-test and post-test measures took place pre-treatment and post-treatment Group therapy was used to encourage participation and model successful mutual support Components of the intervention: Building rapport and trust Cultivating awareness of dysfunctional thoughts and behaviours that are related to their type 2 diabetes Implementing coping skills to control and reduce dysfunctional cognitions and behaviours Cognitive restructuring Stress management Problem-solving Food and HbA1C monitoring (self-monitoring) Relaying information regarding type 2 diabetes and self-management Stress management by keeping a stress log sheet (writing down causes of stress and the coping mechanism used) Homework assignments Social support The control group: No psychological intervention received	Improved glycaemic control A decrease in the emotional burden of managing type 2 diabetes Less physician-related distress Reduced regimen-related distress • Reduced interpersonal distress.
8	Tunsuchart et al. (2020) Benefits of brief group cognitive behavioural therapy in reducing diabetes-related distress and HbA1c in uncontrolled T2DM patients in Thailand *International Journal of Environmental Research and Public Health*	Quasi-experimental pre-test and post-test The study aimed to evaluate how BG-CBT participation affected glycaemic control, diabetes distress, food consumption, physical activity and medication adherence.	Thailand Males and females Aged 18 years or older Experimental group: *n* = 28 Control group: *n* = 28	Diabetes Distress Scale-17, Food Consumption Behaviour Questionnaire (developed by the Bureau of Nutrition, Department of Health, Ministry of Public Health, Thailand), GPAQ version 22, Thai Language Medication-taking Behaviour Scale (MTV-Thai), HbA1c	Nature of the intervention: Brief group cognitive behavioural therapy Six sessions (60 to 90 min) The sessions were held every week The study compared a six-session weekly brief group cognitive behavioural therapy with conventional care control (general counselling) The programme was based on Beck’s work (2011) and was reviewed by a psychiatrist and two psychiatrist nurses (three experts in CBT). The pre-test and post-test measures took place pre-treatment and post-treatment The sessions were led by a psychologist who received training in brief cognitive behavioural group therapy interventions training programme Components of the intervention: Building rapport; psychoeducation regarding the link between triggers, thoughts, emotions, and behaviour Identifying unhelpful and unhealthy behaviour and what triggers it; and modifying behaviours Identifying and linking emotional, cognitive, and behavioural responses Identifying negative automatic thoughts Thoughts that negatively influenced behaviour were adapted Summarising what was learned, developing a plan to monitor goals and achievements The control group: Received no psychological intervention Participants continued with their usual healthcare routine	Reduced HbA1c (improved glycaemic control) Reduced diabetes distress Improved food consumption No statistically significant differences in physical activity and medication adherence
9	Welschen et al. (2013) Effects of a cognitive behavioural treatment in patients with type 2 diabetes when added to managed care; a randomised controlled trial *Journal of Behavioural Medicine*	Randomised controlled trial The research objective was to determine the effect of CBT on the quality of life in individuals with type 2 diabetes.	The Netherlands Males and females Aged between 18 and 75 years The intervention group: *n* = 76 The control group: *n* = 78	Short Questionnaire to Assess Health-enhancing Physical Activity (SQUASH), the DEBQ, The EuroQol (quality of life), CES-D Scale. A questionnaire based on the ASE model was developed to assess behaviour change. Self-reported questionnaires and physical examinations: weight, height, systolic and diastolic blood pressure, HbA1c, levels of total cholesterol, high-density lipoprotein cholesterol, and triglycerides	Nature of the intervention: Cognitive behaviour therapy based on problem-solving training Three to six 30-min sessions Dieticians or diabetes nurses led the sessions depending on the participants’ need The dieticians and diabetes nurses received training in CBT and received two days of training on how to implement the intervention All sessions were recorded, and the nurses and dieticians had supervision by a CBT psychologist every four weeks During the initial session, a diabetes nurse assessed the most critical behavioural domain Should the need have been dietary, the participant was referred to a dietician If it was another domain, a diabetes nurse continued with the intervention Measurements were performed at the start of the intervention, after six months and 12 months HDL cholesterol and triglycerides were measured at baseline and after 12 months Components of intervention: Facilitate decision-making Increase physical activity Change eating behaviours Problem-solving training: Defining the problem; setting goals; identifying solutions; implementing solutions; evaluating the outcomes The control group: Received managed care Managed care included an annual visit to the diabetes care system, which includes consultations with a diabetes nurse and dietician	Short-term effects (between 0 and 6 months): Improved quality of life Additional outcomes that the study reported were: Increased physical activity Decreased depressive symptoms Long term (12 months), the effects were not present It is suggested that more intensive interventions are required

*Source:* Ardeshirpey, J., Bakhshayesh, A.R., Dehghan, M. & Abadi, H.Z.M., 2021, ‘Effect of mindfulness-based cognitive therapy on cognitive emotion regulation, perseverative thinking, and glycemic index in patients with type 2 diabetes – A trial study’, *Iranian Journal of Diabetes and Obesity* 13(3), 150–159. https://doi.org/10.18502/ijdo.v13i3.7189; Empraninta, H.E., Purba, J.M. & Asrizal, 2021, ‘The effect of cognitive behavioural therapy on the self-management and self-care behaviour of type 2 diabetes mellitus patients’, *Jurnal Keperawatan Soedirman* 16(1), 20–24. https://doi.org/10.20884/1.jks.2021.16.1.1569; Inouye, J., Li, D., Davis, J. & Arakaki, R., 2015, ‘Psychosocial and clinical outcomes of a cognitive behavioral therapy for Asians and Pacific Islanders with type 2 diabetes: A randomized clinical trial’, *Hawai’i Journal of Medicine & Public Health* 74(11), 360–368; Mousavian, N., Mujembari, A.K. & Aghayousefi, A., 2018, ‘The effectiveness of cognitive therapy on quality of life in patients with type II diabetes’, *International Archives of Health Sciences* 5(4), 115–119. https://doi.org/10.4103/iahs.iahs_35_18; Pan, X., Wang, H., Hong, X., Zheng, C., Wan, Y., Buys, N. et al., 2020, ‘A group-based community reinforcement approach of cognitive behavioral therapy program to improve self-care behavior of patients with type 2 diabetes’, *Frontiers in Psychiatry* 11, 719. https://doi.org/10.3389/fpsyt.2020.00719; Seyed-Reza, A., Norzarina, M.Z. & Kimura, L.W., 2015a, ‘Effect of group cognitive behavioral therapy (CBT) on psychological well-being and glycemic control in adults with type 2 diabetes’, *International Journal of Diabetes in Developing Countries* 35(2), 284–289. https://doi.org/10.1007/s13410-015-0415-z; Seyed-Reza, A., Norzarina, M.Z. & Kimura, L.W., 2015b, ‘The benefits of cognitive behavioral therapy (CBT) on diabetes distress and glycemic control in type 2 diabetes’, *Malaysian Journal of Psychiatry* 24(2), 18–28; Tunsuchart, K., Lerttrakarnnon, P., Srithanaviboonchai, K., Likhitsathian, S. & Skulphan, S., 2020, ‘Benefits of brief group cognitive behavioral therapy in reducing diabetes-related distress and HbA1c in uncontrolled type 2 diabetes mellitus patients in Thailand’, *International Journal of Environmental Research and Public Health* 17(15), 1–10. https://doi.org/10.3390/ijerph17155564; Welschen, L.M.C., Van Oppen, P., Bot, S.D.M., Kostense, P.J., Dekker, J.M. & Nijpels, G., 2013, ‘Effects of a cognitive behavioural treatment in patients with type 2 diabetes when added to managed care: A randomised controlled trial’, *Journal of Behavioral Medicine* 36(6), 556–566. https://doi.org./10.1007/s10865-012-9451-z

FBS, fasting blood sugar; MBCT, mindfulness-based cognitive therapy; DSMQ, Diabetes Self-management Questionnaire; SDSCA, Summary of Diabetes Self-care Activities; CBT, cognitive behaviour therapy; T2DM, type 2 diabetes mellitus; DQOL, Diabetes Quality of Life; CES-D, Center for Epidemiological Studies Depression; SF-36, 36-item Short Form Health; MDQ, Multidimensional Diabetes Questionnaire; DES, diabetes education and support; W-BQ, Well-Being Questionnaire; DDS, Diabetes Distress Scale; BG-CBT, brief group cognitive behavioural therapy; GPAQ, Global Physical Activity Questionnaire; DEBQ, Dutch Eating Behaviour Questionnaire.

### Ethical considerations

This research study obtained ethics approval from the Health Research Ethics Committee (HREC) of the North-West University (NWU), NWU-00301-21-S1. A rapid review is rigorous and adheres to the core principles of a traditional systematic review to avoid prejudice during the research process (Schünemann & Moja 2015). The research was conducted according to the principles set out by Suri (2020), which included continuously being aware of subjectivity and engaging in reflection regarding the research process. Purposefully informed selective inclusivity was strengthened by recording, documenting, and adhering to the review protocol. Furthermore, trustworthiness and credibility were enhanced by upholding the principles of audience-appropriate transparency, avoiding plagiarism, and circumventing redundant publication (Suri 2020).

## Results

Nine articles published between 2012 and 2022 were identified as eligible for inclusion in the review. More than half the studies were based in developing countries such as Iran (Ardeshirpey et al. 2021; Mousavian et al. 2018), Indonesia (Empraninta et al. 2021), China (Pan et al. 2020), Malaysia (Seyed-Reza et al. 2015a, 2015b), and Thailand (Tunsuchart et al. 2020), while the rest of the studies drew their samples from developed countries such as the United States (specifically Hawaii) (Inouye et al. 2015) and the Netherlands (Welschen et al. 2013). The study conducted in Malaysia published two articles on different aspects of the study (Seyed-Reza et al. 2015a, 2015b).

All the nine articles used quantitative methods (Ardeshirpey et al. 2021; Empraninta et al. 2021; Inouye et al. 2015; Mousavian et al. 2018; Pan et al. 2020; Seyed-Reza et al. 2015a, 2015b; Tunsuchart et al. 2020; Welschen et al. 2013). The quantitative studies mainly employed standardised scales and questionnaires.

**FIGURE 2:**
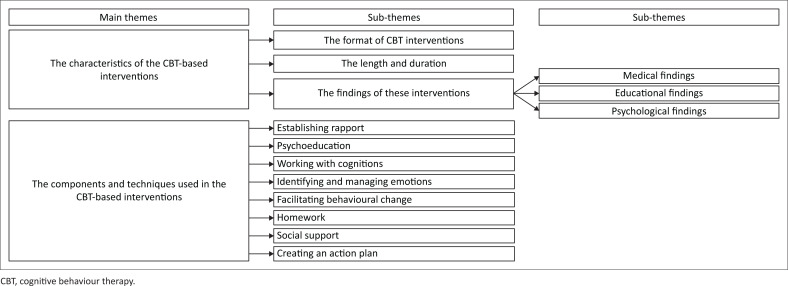
Themes identified.

The themes identified and summarised in Figure 2 are presented according to the aims of the research study:

- The characteristics of the CBT-based interventions.
- The components/techniques used to improve self-management practices.

### The characteristics of the cognitive behaviour therapy-based interventions

The CBT-based interventions varied in format, number of sessions, duration, and the individuals who presented the interventions. Three subthemes emerged from the reviewed articles: (1) the format of CBT interventions, (2) the length and duration of the CBT-based interventions, and (3) the findings of these interventions.

#### The format of the cognitive behaviour therapy-based interventions

Eight of the studies implemented a group-based CBT (Ardeshirpey et al. 2021; Empraninta et al. 2021; Inouye et al. 2015; Mousavian et al. 2018; Pan et al. 2020; Seyed-Reza et al. 2015a, 2015b; Tunsuchart et al. 2020), with the average number of participants in the groups ranging from 10 to 15 participants. Within the group-based intervention, four of the articles used a customary CBT intervention (Empraninta et al. 2021; Inouye et al. 2015; Seyed-Reza et al. 2015a, 2015b).

Ardeshirpey et al. (2021) implemented a mindfulness-based component in their CBT approach, whereas Mousavian et al. (2018) focused on cognitive therapy. Pan et al. (2020) employed a group-based community reinforcement approach (CRA) to CBT. Tunsuchart et al. (2020) and Welschen et al. (2013) employed a brief cognitive behavioural treatment, with Welschen et al. (2013) incorporating problem-solving components.

#### The length and duration of sessions

Cognitive behaviour therapy was conducted weekly and biweekly by psychologists, researchers, research assistants and community-based physicians who were pre-trained in CBT by psychologists, dieticians and diabetes nurses. The total number of sessions ranged from 6 to 12, while the duration was between 30 – 120 min. The average duration of the interventions was 2 to 3 months.

#### The findings of the cognitive behaviour therapy-based interventions

Three subthemes regarding the findings of CBT-based interventions were identified: (1) medical findings, (2) educational findings, and (3) psychological findings.

The long-term interventions were linked to positive findings (Ardeshirpey et al. 2021; Mousavian et al. 2018; Pan et al. 2020; Seyed-Reza et al. 2015a, 2015b), with both Mousavian et al. (2018) and Pan et al. (2020) reporting continuous positive effects after their follow-up of 3 and 6 months respectively. Notably, Inouye et al. (2015) and Welschen et al. (2013), whose interventions were shorter in duration, reported no significant effects present after a 12-month post-intervention follow-up, suggesting the need for more intensive and lengthier CBT interventions.

**Medical findings:** Five of the studies that implemented a CBT-based intervention reported that the participants in the experimental groups experienced improved glycaemic control (Ardeshirpey et al. 2021; Inouye et al. 2015; Pan et al. 2020; Seyed-Reza et al. 2015b; Tunuchart et al. 2020).

**Educational findings:** Improved knowledge, attitude and behaviours towards the self-management of T2DM were reported by Ardeshirpey et al. (2021), Empraninta et al. (2021), Inouye et al. (2015), Mousavian et al. (2018), Seyed-Reza et al. (2015a) and Pan et al. (2020). Psychoeducation was used to educate participants regarding maladaptive cognitive processes and how they influence self-management practices.

**Psychological findings:** This subtheme included increased quality of life and emotional regulation.

The studies performed by Inouye et al. (2015), Mousavian et al. (2018) and Seyed-Reza et al. (2015a) found an overall increase in psychological well-being and quality of life because of participants experiencing enhanced self-efficacy, concentration, problem-solving and flexibility in their self-management practices (Welschen et al. 2013).

Ardeshirpey et al. (2021), Seyed-Reza et al. (2015a) and Tunsuchart et al. (2020) found that CBT-enabled participants could learn how to identify, monitor, endure and manage their emotions, which ultimately improved emotional regulation skills.

### The components and techniques used in the cognitive behaviour therapy-based interventions

Eight subthemes emerged regarding the techniques and components that were used in the CBT-based interventions: (1) establishing rapport, (2) psychoeducation, (3) working with cognitions, (4) identifying and managing emotions, (5) facilitating behavioural change, (6) social support, (7) homework, and (8) creating an action plan.

#### Establishing rapport

The CBT-based interventions applied by Ardeshirpey et al. (2021), Mousavian et al. (2018), Pan et al. (2020), Seyed-Reza et al. (2015b) and Tunsuchart et al. (2020) emphasised the principle of building rapport with the participants to maintain an alliance throughout the intervention. Establishing rapport generated trust between individuals, which led to improved treatment outcomes, adherence and self-efficacy.

#### Psychoeducation

The CBT-based interventions conducted by Ardeshirpey et al. (2021), Empraninta et al. (2021), Mousavian et al. (2018), Pan et al. (2020), Seyed-Reza et al. (2015b) and Tunsuchart et al. (2020) included a psychoeducation component. Psychoeducation was used to explain the premise of CBT, as it educated the participants on the relationship between stimuli, cognitions, emotions and behaviours (Ardeshirpey et al. 2021; Tunsuchart et al. 2020). With regard to the self-management of T2DM, the psychoeducation component aimed to explore and explain cognitions, emotions and behaviours, and how these cognitions play a role in everyday self-management practices, beliefs about self-efficacy, glycaemic control and developing coping skills (Ardeshirpey et al. 2021; Pan et al. 2020; Seyed-Reza et al. 2015a).

Furthermore, Pan et al. (2020) and Seyed-Reza et al. (2015b) included an educational component regarding T2DM in their interventions. Information sharing (including educational documents about T2DM) and consultations with general practitioners, information regarding healthy eating habits and nutritional information increased understanding and assisted participants in restructuring their thoughts and generating alternative thoughts regarding T2DM (Seyed-Reza et al. 2015b).

#### Working with cognitions

Different cognitive mechanisms were utilised in the CBT interventions to possibly improve self-management of T2DM and included: (1) fostering cognitive awareness, (2) cognitive restructuring, and (3) cognitive acceptance:

**Fostering cognitive awareness:** Fostering awareness regarding triggers, cognitions, behaviours and emotions was an essential component during the initial sessions of the interventions implemented by Empraninta et al. (2021), Mousavian et al. (2018), Pan et al. (2020), Seyed-Reza et al. (2015a, 2015b), Tunsuchart et al. (2020) and Welschen et al. (2013). Once the participants received psychoeducation regarding triggers and automatic negative thoughts, they were facilitated to decrease dysfunctional thoughts and, in turn, improve decision-making, coping skills and distress tolerance (Mousavian et al. 2018; Seyed-Reza et al. 2015a, 2015b; Tunsuchart et al. 2020; Welschen et al. 2013). The CBT interventions implemented by Ardeshirpey et al. (2021) and Mousavian et al. (2018) educated the participants on cognitive distortions. These included, but were not limited to, catastrophising, generalisation, labelling and prediction (Ardeshirpey et al. 2021).

**Cognitive restructuring:** Once participants could identify dysfunctional thoughts regarding self-management, they could start practising cognitive restructuring (Ardeshirpey et al. 2021; Empraninta et al. 2021; Inouye et al. 2015; Mousavian et al. 2018; Pan et al. 2020; Seyed-Reza et al. 2015a, 2015b; Tunsuchart et al. 2020). Cognitive restructuring uses cognitive reflection to identify automatic negative thought processes and create alternative, flexible thought processes. This is followed by identifying and practising more constructive cognitive and behavioural responses such as normalising mistakes, identifying alternative thoughts and practising self-compassion to reduce negative thoughts and increase realistic decision-making (Mousavian et al. 2018; Seyed-Reza et al. 2015b).

Socratic questioning was used in the interventions implemented by Mousavian et al. (2018) and Pan et al. (2020) and used a set of questions to help participants develop self-reflection and self-monitoring skills.

**Cognitive acceptance:** Ardeshirpey et al. (2021) promoted cognitive acceptance instead of restructuring. Mindfulness-based cognitive therapy (MBCT) focuses on the relationship that participants have with their thoughts rather than modifying the content of their cognitions. In this study, participants were taught to observe and monitor their thoughts to tolerate a range of experiences and unpleasant thoughts without emotional distress. The participants practised decentralisation by viewing thoughts as fleeting and not factual. It emphasised a judgement-free observation of thoughts that promoted endurance instead of avoidance. Awareness of the present was cultivated using techniques such as redirecting awareness to the present, paying attention to the body, and mindful breathing to reduce automatic negative thoughts about the self.

#### Identifying and managing emotions

Emotional regulation was employed by Ardeshirpey et al. (2021), Mousavian et al. (2018), Pan et al. (2020), and Seyed-Reza et al. (2015b). The burden of self-management can cause distressing emotions; therefore, it was important for participants to identify emotional responses and practise emotional regulation skills. Emotional regulation incorporates a cognitive component, as the cognitive interpretation of a situation influences an individual’s emotional reactions (Ardeshirpey et al. 2021). The sessions in the CBT intervention implemented by Ardeshirpey et al. (2021) guided participants to regulate emotions by identifying, monitoring, evaluating and modifying the emotions experienced. This was achieved by practising meditation, learning grounding techniques, doing breathing exercises, fostering awareness of emotions, practising being present, identifying pleasant situations and doing body scans (check-ups). Similarly, Inouye et al. (2015) incorporated biofeedback-assisted relaxation techniques.

#### Facilitating behaviour change

Behaviour change was targeted in the interventions conducted by Inouye et al. (2015), Mousavian et al. (2018), Pan et al. (2020), Seyed-Reza et al. (2015b), Tunsuchart et al. (2020) and Welschen et al. (2013). It included behaviour modification, behaviour rehearsal and enhancing coping skills. Pan et al. (2020) targeted specific behaviour change. Behaviour modifications included analysing sleep patterns, quitting smoking, changing unhealthy eating patterns, increasing physical activity, and reducing alcohol intake (Pan et al. 2020). Positive reinforcement was implemented, and different coping strategies were explored and administered (Pan et al. 2020).

Cultivating these behavioural skills enabled the participants to have more flexible lifestyles and achieve a better quality of life, both associated with diabetes self-management (Mousavian et al. 2018).

#### Homework

Ardeshirpey et al. (2021), Mousavian et al. (2018), Inouye et al. (2015), Pan et al. (2020) and Seyed-Reza et al. (2015b) assigned homework at the end of the sessions during the interventions. The homework assignment depended on the content covered in each session and was used to reinforce what was learned during the sessions (Pan et al. 2020). Homework tasks included practising the learned behaviour outside the sessions (Inouye et al. 2015); providing feedback; completing log sheets to track triggers, coping skills and the efficacy of coping skills (Seyed-Reza et al. 2015b); diary writing; monitoring challenges and symptoms; and implementing self-care skills (Pan et al. 2020).

Self-monitoring formed part of homework assignments (Inouye et al. 2015; Pan et al. 2020; Seyed-Reza et al. 2015a, 2015b). Participants monitored and recorded their HbA1C levels, level of anxiety, food intake, medication adherence and sleeping patterns (Pan et al. 2020; Seyed-Reza et al. 2015a, 2015b), which enabled them to connect the different components, form an action plan, take responsibility for implementing change, and reduce distress.

#### Social support

Social support was evident in the group-based interventions (Ardeshirpey et al. 2021; Inouye et al. 2015; Mousavian et al. 2018; Pan et al. 2020; Seyed-Reza et al. 2015a, 2015b; Tunsuchart et al. 2020). A group setting enabled participants to disclose their experiences and opinions. The group context facilitated social support, the modeling of successful self-management, accountability and social reinforcement. Mutual support and positive reinforcement positively affected glycaemic control (Inouye et al. 2015; Mousavian et al. 2018; Pan et al. 2020; Seyed-Reza et al. 2015a, 2015b; Tunsuchart et al. 2020).

In addition, sharing similar experiences and facing similar challenges created an environment where there was less stigmatisation and normalised some of the experiences and difficulties that the participants faced (Tunsuchart et al. 2020). The main advantages of group-based CBT interventions were summarised as catharsis, universalisation, imparting knowledge, providing feedback and altruism (Seyed-Reza et al. 2015b).

#### Creating an action plan

At the end of the intervention, time was taken to summarise the knowledge and skills learned during the sessions (Ardeshirpey et al. 2021; Mousavian et al. 2018; Tunsuchart et al. 2020). Plans were developed among the participants and presenters for future challenges and changes. This included monitoring and acknowledging the achievement of goals, implementing learned skills daily and using the knowledge obtained to cope with future difficulties.

## Discussion

The rapid review aimed to identify CBT interventions and techniques that address self-management practices for individuals with T2DM.

Firstly, functional characteristics of CBT interventions were identified that could potentially be of value in developing countries. Interventions were mainly group-based and promoted peer social support, which was recognised as one of the most critical components that facilitated self-management (Seyed-Reza et al. 2015b). Social support, specifically social inclusion, has been linked to improved health outcomes such as improved glycaemic control and low-density lipoprotein (McCoy & Theeke 2019). McCoy and Theeke (2019) concluded that social support and inclusion are essential for coping with T2DM. Future interventions will benefit by including this component, as it aids with the self-management of T2DM. Burlingame et al. (2016) conducted a meta-analysis that found no difference in treatment outcomes between individual and group formats. Although the therapeutic factors, such as social support in a group setting (Johnsen & Timm 2018), may differ, individual and group formats share the same treatment techniques and philosophy. Therefore, the environment, the needs of the individuals and socioeconomic factors must be considered when choosing an individual or group-based intervention.

Secondly, the duration of interventions was an important factor to consider, with longer-term interventions yielding better outcomes. However, considering the context of many developing countries, including the limited access to healthcare in South Africa, the number of sessions included in the intervention must maintain efficacy and consider the accessibility of treatment (Misra et al. 2019). Brief CBT is approximately six to eight sessions (Cully et al. 2020), and the number of sessions depends on various factors, namely the setting, the intervention provider, and the client. A study by Bortoncello et al. (2022) found no significant effect between fewer or more sessions (approximately seven to 14 sessions) and the duration of the sessions. Sessions that lasted between 90 – 120 min showed the same effect. The advantages of a briefer intervention include being more cost-effective and accessible, improved credibility and enhanced patient motivation (Curwen, Palmer & Ruddel 2000).

Cully et al. (2020) argue that if a briefer intervention is considered, specificity is required in treatment, and the client needs to take responsibility for extra reading and homework assignments. Considering the findings of the brief CBT that was implemented by Inouye et al. (2015) and Welschen et al. (2013) and the recommendation for more intensive interventions, the theme that arises is that an intervention can be from 8 – 12 sessions and conducted on a weekly to a biweekly basis for approximately 90 min, as displayed by the studies in this review.

Thirdly, it was essential to note the setting in which these studies took place, how these settings relate to T2DM, and why the setting emphasises the need for interventions. Seven studies were conducted in developing countries (The Investopedia Team 2022). Misra et al. (2019) suggested using the community of health providers to increase knowledge and provide interventions for T2DM. This is supported in the review, as many individuals who presented the interventions received training before implementing the intervention. This training was presented by researchers, research assistants, community-based physicians (trained by psychologists), psychologists, dieticians, and diabetes nurses. The interventions were then more accessible to individuals in developing countries.

As a developing country, South Africa faces similar challenges, such as limited funding, restrained resources and inadequate access to healthcare professionals. Therefore, exploring interventions that can be implemented within various health settings and access a larger community is crucial in the South African context.

After considering the format, duration and setting, the components included under the umbrella of CBT-based interventions were crucial to determine whether there was a synthesis of techniques that facilitated self-management practices.

The techniques and components identified in the different reviews reflected a fundamental goal in CBT, namely developing a repertoire of skills to alter maladaptive cognitive and behavioural patterns (Yang et al. 2020). The findings of the rapid review suggest that the techniques identified were effective in helping participants to gain control over psychological and physical symptoms, which is also reported by Andreae et al. (2020). Many of the studies in the review employed what is considered to be these traditional CBT techniques (Wolgast, Lundh & Viborg 2013). The cognitive acceptance, which forms part of the third wave of CBT, was implemented in one of the studies (Wolgast et al. 2013). Cognitive acceptance stresses the relationship with thoughts and is linked to mindfulness. Cognitive acceptance has also been linked to improved disease self-management (Goodwin et al. 2011) and therefore needs to be considered an essential component of the CBT intervention. This repertoire of self-management practices and skills that CBT-based interventions facilitate has proven to increase active participation and involvement with the self-management of T2DM (Koochaksaraee et al. 2022). Therefore, it can be deemed vital that these components and techniques are thoroughly considered and included in CBT-based interventions.

## Limitations

Only five databases were searched. Some articles therefore may not have been included. Only nine articles on CBT-based interventions for T2DM self-management were included in the review of diabetes. Caution should therefore be exercised when concluding the effectiveness of CBT-based interventions for self-management.

The studies were heterogeneous in quality and methodological approach, and no studies were conducted in South Africa. South Africa is considered a developing country and many studies were conducted in developing countries. However, it would be advisable to consider South Africa’s cultural context before generalising the findings.

## Conclusions and future directions

This study emphasised the need for further comprehensive research to evaluate CBT-based interventions for the self-management of T2DM. As a result of the small, heterogeneous selection of studies, only a few general trends can be highlighted. Although the review found that CBT-based interventions were linked to positive findings in terms of self-management, further comparative studies are required to measure the effectiveness of this approach in different contexts, especially in developing countries.

Cognitive acceptance was a therapeutic intervention that requires further investigation. Exploring cognitive awareness in relation to the self-management of T2DM can provide insight into how this relationship with distressing thoughts facilitates self-management.

Lastly, the reviewed articles do not provide much information on CBT-based interventions for self-management in a South African context. It is suggested that socio-economic disadvantages and accessibility to such interventions should be considered in future research.

The following areas can be explored in future research:

- Future studies can include a follow-up or more extended follow-up period to determine the longevity of the outcomes and the effectiveness of the interventions.
- Studies should focus on individual and group-based CBT interventions to determine the effectiveness of these formats for the improvement of self-management.
- More CBT-based interventions for improved self-management in the South African context can be conducted.
